# Experiences of adult cancer survivors in transitions

**DOI:** 10.1007/s00520-018-4605-3

**Published:** 2018-12-26

**Authors:** Margaret Fitch, Sarah Zomer, Gina Lockwood, Cheryl Louzado, Raquel Shaw Moxam, Rami Rahal, Esther Green

**Affiliations:** 1 Bloomberg Faculty of Nursing, University of Toronto, Toronto, Canada; 2 Canadian Partnership Against Cancer, Toronto, Canada

**Keywords:** Cancer survivors, Transitions in care, Integrated care, Survivorship care, Follow-up care

## Abstract

**Purpose:**

To understand the experiences of adult cancer survivors as they transition from the end of cancer treatment to follow-up care as a basis for developing actionable recommendations to integrate cancer care delivery and survivorship care.

**Methods:**

A national survey was conducted in collaboration with ten Canadian provinces to identify unmet needs and experiences with follow-up for cancer survivors between 1 and 3 years post-treatment. Surveys were available in English and French and completed either on paper or on-line. Samples were drawn from provincial cancer registries and packages distributed by mail.

**Results:**

A total of 40,790 survey packages were mailed out across the ten provinces and 12,929 surveys were completed by adults (age 30+ years), and 329 surveys were completed by adolescents and young adults (age 18 to 29 years) giving an overall response rate of 33.3%. For the purposes of this publication, the focus will be on the adult sample. In the adult cohort (age 30+ years), 51% of the sample were females, 60% were 65 years of age or older, and 77% had not experienced metastatic spread. Three-quarters reported their health as good/very good and 82% that their quality of life was good/very good. Overall, 87% experienced at least one physical concern, 78% experienced at least one emotional concern, and 44% experienced at least one practical concern. The average number of concerns reported for each domain ranged from 2.0 to 3.8. For those who sought help, a third experienced difficulty obtaining assistance or did not receive it. The most frequently cited reasons for not seeking help was that someone had told them what they were experiencing was normal.

**Conclusions:**

The results indicate that many adult survivors have concerns about physical, emotional, and practical issues but are not receiving help to reduce their suffering. It is imperative we take action to correct this current reality.

## Introduction/purpose

Improved outcomes of cancer treatment have resulted in an ever-growing number of individuals who are living with and beyond cancer. There are more than 1.6 million Canadians who have had a cancer diagnosis and are living after treatment [1].

Cancer survivors experience varied and substantial impacts on their quality of life [2–6]. Fatigue, cognitive changes, lymphedema, and pain are frequently reported [7–9]. Additionally, emotional distress emerges from living with uncertainty, finding a ‘new normal’, returning to work or school, and fear of recurrence [10–14]. Many face significant financial challenges [15] and are more vulnerable to health-related concerns than their healthy counterparts [16].

Relatively little research has been conducted to understand the experiences of survivors in accessing care following the completion of treatment [17] although several recent studies have moved in this direction in Australia [18], United Kingdom [19], United States [3, 20], Denmark [21], and the Asia Pacific Region [22]. These studies described significant survivor concerns, many of which remained unaddressed, and difficulties regarding access, professional responsiveness, co-ordination, communication, and involvement in care.

To date, one Canadian study explored cancer survivors’ needs [23] but experiences were not investigated and the sample was small (*n* = 550). The growing cadre of cancer survivors has stimulated interest in finding care models whereby the responsibility for survivorship care/follow-up care is transitioned from specialist to primary care providers [24]. However, concerns have been voiced that this trend could increase gaps in survivorship care [25, 26].

The Canadian Partnership Against Cancer (the Partnership) held consultations in 2014 with over 75 experts on survivorship, measurement, and cancer system planning. Participants identified transitions, integration, and communication as key areas for survivorship improvements but limited data on follow-up and transitions between care providers was a significant gap. Given these observations, the Partnership undertook a national survey to understand the experiences of cancer survivors as they transition from cancer treatment to survivorship care/follow-up care. Survivorship care or follow-up care is defined as care given to a patient after finishing cancer treatment and prior to identification of recurrent disease. The findings would be a basis for developing actionable recommendations on integrating cancer and survivorship care, ultimately improving the experience of survivors.

## Methods

### Study design and participant eligibility criteria

A survey, *Experience of Cancer Patients in Transition Study* (‘Transitions Study’), was administered across ten Canadian Provinces. The survey focused on cancer survivors between 1 and 3 years following cancer treatment. The population and eligibility criteria included adult survivors (age 30+ years) of breast, prostate, colorectal, and melanoma diseases with no metastatic spread, and selected hematological (e.g., Hodgkin’s lymphoma, diffuse B cell lymphoma, acute myelogenous leukemia, acute lymphocytic lymphocyte leukemia) cancers; and adolescents and young adults (AYA, 18 to 29 years) with all non-metastatic cancer types except testes, where metastatic disease was included. For the purposes of this first paper from the Transitions Study, and due to the magnitude of the dataset, the focus will be on the adult population.

### Governance structure

The Partnership collaborated with ten provincial cancer agencies/programs in this study. Each agency/program appointed a primary investigator and research coordinator for provincial planning, survey dissemination, and national agency interaction. A national Expert Panel provided overall advice with short-term working groups formed as required. The Partnership hired a vendor to assist with survey design, distribution, and analysis.

### Survey development

The survey was designed to answer the following questions:

- What are the needs of cancer survivors 1–3 years after treatment (physical, emotional, practical, and informational)? What are the biggest unmet needs?
- Who are the most vulnerable cancer survivors? What personal characteristics are associated with unmet needs/poor outcomes?
- What factors/system resources are correlated with needs being met? Enablers/predictors of needs being met/positive outcomes achieved?

**Fig. 1:**
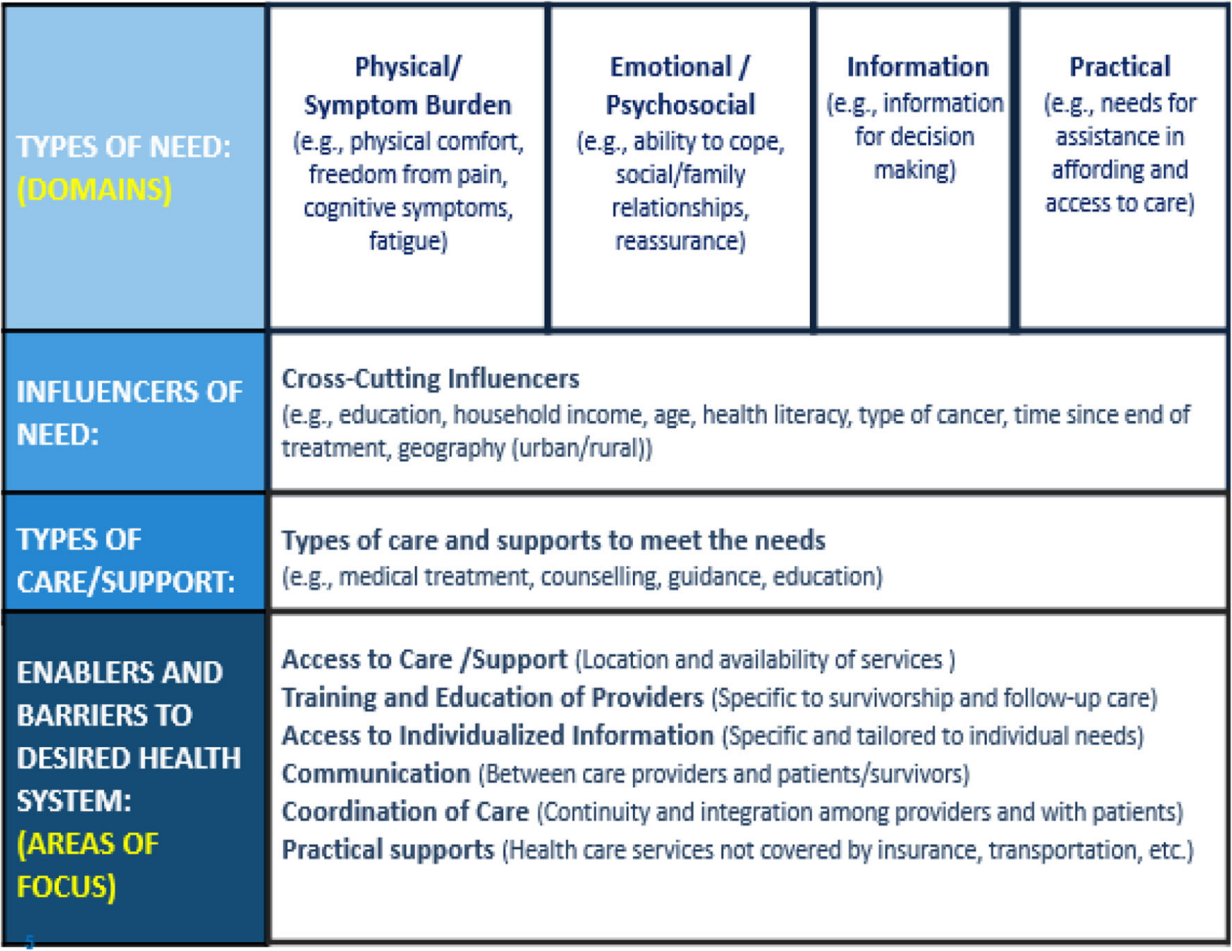
Conceptual framework for transitions study

A literature review was conducted in 2015 to inform the conceptual framework for the survey [27, 28], to direct subsequent consultations and guide the development of survey items. Both peer-reviewed and gray literature were accessed to identify existing research on patient/survivor needs, patient/survivor experience, and relevant survey instruments. The databases included in the search were PubMed, PsychInfo, Medline, ProQuest Nursing and Allied Health, JSTOR, Web of Science, Science Direct, CINAHL, Google Scholar, JBI, and the Cochrane Library. Consultations were held with cancer survivors (11 adults, 4 AYA), clinicians (12), and system leaders (8) to gather feedback about the framework’s relevance (Fig. 1). The survey items were crafted based on the framework, consultations, review of existing surveys, and input from key stakeholder groups, through an iterative process. Once finalized, the survey was translated into French using a process that ensured content and semantic equivalence. An on-line survey version was designed in both languages.

Cognitive interviews (60–90 min) were completed with 15 different cancer survivors who met the eligibility criteria to evaluate the survey’s meaningfulness, clarity, understandability, and ease of completion. The survey underwent performance testing with 96 survivors, recruited to match the eligibility criteria. Only small adjustments to the survey were made following these evaluations.

The final version contained closed and open-ended items organized as about you, your health and well-being, about your history with cancer, health care providers who oversee your follow-up cancer care, overall experiences with follow-up, understanding changes in your life, access to follow-up cancer care plans/medical records, health insurance, socio-demographic questions, Internet use, and final comments. The paper survey took about 45 min to complete and the online version took 30 min. The ten provinces submitted to ethics and privacy approvals prior to data collection. A copy of the survey is available on the Canadian Partnership Against Cancer System Performance site http://www.systemperformance.ca/transition-study/

### Sample selection

Provincial staff drew eligible patients from provincial cancer registries based on eligibility criteria and linking to treatment data to confirm that treatment had occurred and end of treatment dates (defined for each disease type). The number of surveys was calculated for the adult sample such that 95% confidence intervals, for a percentage assumed to be 50%, would have width no more than ± 5% by disease site and province and assumed a response rate of 30%. Estimates of the number of eligible survivors for each province were based on disease site prevalence at the national level and incidence by province. As smaller provinces were not likely to have enough survivors within disease sites to achieve desired confidence interval precision, all eligible survivors were surveyed. For larger provinces and disease sites where the number of eligible survivors was anticipated to be greater than the required number, a random sample within the cancer disease site was chosen. The sample would be sufficient to achieve precision at least ± 3% by disease site for all provinces combined. A total of 40,790 survey packages were mailed out across the 10 provinces.

### Survey dissemination

The survey was disseminated through each provincial cancer agency/registry and customized as required. Each province mailed a survey package to the survivor’s residence to survivors drawn from the registry database. All recipients, except in Quebec, had the option of completing the survey online. One reminder letter was sent to those who had not responded approximately 4 weeks after the initial mailing. Quebec utilized telephone call reminders.

The survey package contained (1) a cover letter (customized to provincial requirements with brief study description, confidentiality explanations, URL link and PIN to the encrypted online survey, consent documents in Ontario and Quebec); (2) paper survey copy with pre-printed barcode and associated PIN (for those who preferred a paper version); and (3) pre-addressed, pre-paid return envelope. Ontario differed from all provinces by using a double consent process.

The survey was offered in English across all provinces except New Brunswick and Quebec. New Brunswick mailed English and French survey versions to all recipients. Quebec recorded language preferences in their sample pull and mailed roughly 25% in English. However, any recipient across the country could request a French survey version.

The Research Coordinators managed project-specific provincial phone lines to provide assistance to respondents. Data were collected in 2016 in parallel for 18–19 weeks in 7 provinces, 8 weeks in Quebec, 9 weeks in British Columbia, and 15 weeks in Ontario.

### Analysis

This manuscript is focused on the initial question regarding unmet needs for the adult respondents. Data were analyzed using SAS 9.4. Descriptive statistics were used to summarize data. The frequency of individual needs was calculated separately within each domain (physical, emotional, practical) and the corresponding level of concern and help-seeking for each need determined by crosstabs. Concerns identified by respondents in the ‘*big*’ category were treated as ‘severe’ and ‘difficulty getting help’ included responses ‘hard’, ‘very hard’, and ‘did not get help’. ‘Unmet need’ was defined as the percentages of respondents who did not receive help regardless of whether or not they sought help. Unmet needs were rank-ordered by the number who experienced a concern (magnitude of concern) and the percentages of those respondents who did not receive help. The frequency of response for each informational item was calculated. Because of the different format for these items, ‘*unmet need*’ included the negative responses (i.e., ‘disagree’, ‘strongly disagree’) and percentages of negative responses were rank-ordered.

### Sample limitations

Confidentiality issues limited information about characteristics of survivors that could be shared with the vendor. As a result, there was insufficient detail to allow weighting of surveys to make them representative of all Canadian survivors. Further, although the intention of sampling was to target five disease sites and survivors 1–3 years post-treatment, self-reported survey data revealed that just under 10% of survivors indicated they had a cancer site outside the five targeted, and only 48% reported being between 1 and 3 years post-treatment. Analysis of missing data patterns revealed that data were not missing completely at random, emphasizing that this sample should not be generalized to represent all survivors in Canada. Unweighted data from all survey respondents are presented.

## Results

### Sample-selected demographics

A total of 40,790 survey packages were mailed out across the ten provinces and 12,929 surveys were completed by adults (age 30+ years), and 329 surveys were completed by adolescents and young adults (18 to 29 years) giving an overall response rate of 33%. The final sample includes all respondents who returned a paper copy by mail or accessed the survey on-line and completed the initial demographic information. Eighty-two percent (82%) of the respondents returned their survey by mail.

Of the adult sample, 51% were females and 60% were 65 years of age or older (see Table 1). Ninety-two percent reported that their diagnosis occurred between 2012 and 2014 and 77% indicated that they had not experienced metastatic disease. Breast cancer (29%) and prostate cancer (24%) survivors constituted the largest respondent groups. Of note, 14% of the respondents reported having more than one cancer and 65% reported experiencing co-morbid conditions (i.e., cardiovascular or hypertension = 30% and arthritis or rheumatic disease = 28%). The majority received some form of treatment (e.g., surgery, radiation, chemotherapy, hormonal therapy) with 4% indicating that they had not received cancer treatment. Approximately 72% indicated their last treatment occurred between 1 and 5 years ago. Overall, three-quarters of the respondents reported that their physical and emotional health was good/very good and 82% rated their quality of life as good/very good.

**Table 1:** Description of the adult survey sample (*N* = 12,929)

Characteristic	*N*	%
Sex		
- Male	6272	48.5
- Female	6614	51.2
- Prefer not to answer	43	0.3
Age		
- 30-54	1802	13.9
- 55-74	7853	60.7
- 75 and over	3274	25.3
Marital status		
- Single	765	5.9
- Married/partnered	9570	74.0
- Separated/divorced/widowed	2487	19.2
- Prefer not to answer	107	0.8
Education		
- High school or less	7358	56.9
- Post-secondary degree (college/university)	3959	30.6
- University graduate degree	1097	8.5
- Missing	515	4.0
Income		
-< $25,000	1643	12.7
- $25,000 to < $50,000	3019	23.4
- $50,000 to < $75,000	2094	16.2
- $75,000 or more	3126	24.2
- Prefer not to answer	2567	19.9
- Missing	480	3.7
Employment		
- Employed (full time, part time, paid leave)	4010	31.0
- Not in paid employment (homemaker, student, retired)	8148	63.0
- Unemployed	296	2.3
- Missing	475	3.7
Place of residence		
- Rural (community < 10,000)	4564	35.3
- Urban (community ≥ 10,000)	7965	61.6
- Missing	400	3.1
Disease site^a^		
- Breast	3751	29.0
- Prostate	3064	23.7
- CRC	2576	19.9
- Hematological	1077	8.3
- Melanoma	1494	11.6
- Other	464	3.6
- Missing	777	6.0
Metastases		
- No metastases	9906	76.6
- Living with metastases	1175	9.1
- Unsure	1098	8.5
- Missing	750	5.8
Time since treatment		
- < 1 year	1483	11.5
- 1 year to < 3 years	5960	46.1
- 3 years or more	3127	24.2
- Did not receive treatment	1851	14.3
- Missing	508	3.9
Type of treatment received		
- Surgery only	3602	27.9
- Drug therapy only (chemo/non-chemo)	968	7.5
- Radiation therapy only	1321	10.2
- Combination therapy/other	5957	46.1
- No treatment/no plan for tx/active surveillance	548	4.2
- Missing	533	4.1
Comorbidities (4 most common)		
- Cardiovascular or heart condition; hypertension or high blood pressure	3863	29.9
- Arthritis, osteoarthritis, or other rheumatic disease	3569	27.6
- Diabetes	1539	11.9
- Mental health issues	1232	9.5
General physical health		
- Very poor/poor	488	3.8
- Fair	2841	22.0
- Good/very good	9539	73.8
- Missing	61	0.5
General emotional health		
- Very poor/poor	452	3.5
- Fair	2256	17.5
- Good/very good	9617	74.4
- Missing	604	4.7
Overall quality of life		
- Very poor/poor	268	2.1
- Fair	2007	15.5
- Good/very good	10,619	82.1
- Missing	35	0.3

^a^Percentages add up to more than 100% because patient/survivors can be included in more than one site if they chose two or more. The Other category contains those who chose a site (one or multiple) that was not one of the five shown here

### Physical changes

The majority of respondents (87%) experienced at least one physical symptom while 58% experienced 3 or more. On average, 3.8 physical symptoms were reported by those with at least one symptom. The most common symptoms across all cancer sites were fatigue/tiredness (67%), change in sexual function/activity (44%), and change in memory/concentration (39%), and nerve problems (36%) (see Table 2).

**Table 2:** Prevalence of physical symptoms and access to help in adult survey sample

Physical Symptoms	Number of respondents who answered question	Number of respondents indicating a concern about a physical symptom (mild, moderate, or big)	% of those experiencing a concern about a physical symptom whose concern was ‘*bi*g’	% of those experiencing concern about a physical symptom whose concern was ‘*moderate*’	% of those with a concern about a physical symptom who sought help^a^	% of those who sought help for their concern that experienced difficulty (hard or very hard to find help/no help obtained)^a^
Fatigue or tiredness	12,021	8102 (67%)	2678 (33%)	3231 (40%)	2975 (38%) *N* = 7748	1093 (37%) *N* = 2944
Changes in sexual activity or function	11,967	5321 (44%)	2375 (45%)	1653 (31%)	2042 (40%) *N* = 5124	740 (36%) *N* = 2028
Changes to concentration or memory	11,877	4593 (39%)	970 (21%)	1627 (35%)	1188 (27%) *N* = 4369	564 (48%) *N* = 1175
Nerve problems	11,868	4326 (36%)	1087 (25%)	1481 (34%)	2230 (54%) *N* = 4168	877 (40%) *N* = 2211
Gastrointestinal problems	11,889	4292 (36%)	1242 (29%)	1556 (36%)	2617 (63%) *N* = 4131	699 (27%) *N* = 2596
Bladder or urinary problems	11,935	4156 (35%)	1140 (27%)	1486 (36%)	2334 (58%) *N* = 3991	505 (22%) *N* = 2314
Chronic pain or long term pain	11,819	4014 (34%)	1046 (26%)	1442 (36%)	2363 (61%) *N* = 3867	829 (35%) *N* = 2341
Hormonal, menopause or fertility	11,632	2947 (25%)	1102 (37%)	1009 (34%)	1466 (51%) *N* = 2847	505 (35)% *N* = 1453
Lymphedema	11,771	2699 (23%)	696 (26%)	936 (35%)	1671 (63%) *N* = 2636	397 (24%) *N* = 1659

^a^Note that only those who answered relevant question(s) were included. N refers to the denominator for each concern

Of individuals who were concerned about a physical symptom, the highest percentage who expressed having a ‘big’ concern cited changes in sexual function/activity (45%), hormonal/menopause or fertility (37%), and fatigue/tiredness (33%). Gastrointestinal problems (63%) and pain (61%) were the symptoms for which respondents most frequently sought help. However, more than one-third of respondents who sought help indicated that it was difficult to obtain for most of their symptoms. Seeking help for changes in concentration and memory was particularly challenging (48% experienced difficulty).

Of those who did not seek help, 33% indicated that they did not seek help because someone had told them it was normal to expect and they did not think anything could be done. Ten percent said they did not want to ask for help.

### Emotional changes

The majority of respondents (78%) experienced at least one emotional issue with 42% experiencing 3 or more. On average, 3.3 emotional issues were reported by those with at least one concern (see Table 3). Many survivors (68%) reported suffering from anxiety and worry about cancer recurrence. Additionally, 42% experienced changes in sexual intimacy and 46% reported changes in depression, sadness, and loss of interest in daily activities.

**Table 3:** Prevalence of emotional issues and access to help in adult survey sample

Emotional issues	Number of respondents who answered question	Number of respondents indicating a concern about an emotional issue (mild, moderate or big)	% of those experiencing a concern about an emotional issue whose concern was ‘*bi*g’	% of those experiencing concern about an emotional issue whose concern was ‘*moderate*’	% of those with a concern about an emotional issue who sought help^a^	% of those who sought help for their concern that experienced difficulty (hard or very hard to find help/no help obtained)^a^
Anxiety, stress, worry about cancer returning	11,309	7657 (68%)	1825 (24%)	2663 (35%)	2278 (31%) *N* = 7341	509 (22%) *N* = 2264
Depression, sadness, loss of interest in everyday things	11,038	5106 (46%)	1093 (21%)	1865 (37%)	1677 (34%) *N* = 4964	405 (24%) *N* = 1666
Changes in sexual intimacy	12,043	5025 (42%)	1879 (37%)	1571 (31%)	1468 (31%) *N* = 4786	544 (37%) *N* = 1452
Changes in body image (i.e., confidence in appearance, etc.)	12,093	4670 (39%)	1111 (24%)	1459 (31%)	979 (22%) *N* = 4385	369 (38%) *N* = 971
Changes in relationship with family, partners	12,141	3879 (32%)	840 (22%)	1319 (34%)	784 (21%) *N* = 3715	277 (36%) *N* = 775
Changes in relationship with friends or co-workers	12,082	2523 (21%)	353 (14%)	777 (31%)	338 (14%) *N* = 2372	139 (41%) *N* = 337

^a^Note that only those who answered relevant question(s) were included. N refers to the denominator for each concern

Of those who experienced concerns, 37% identified changes in sexual intimacy of greatest concern. About a third of survivors sought help regarding sexual intimacy (31%), stress (31%), or depression (34%). Of those who did seek help, about 25% experienced difficulty in obtaining assistance or did not receive it.

Ninety-four percent of those with emotional concerns indicated the reason they did not ask for help; 22% indicated that someone had told them it was normal to experience emotional concerns, or they did not want to ask (18%).

### Practical challenges

Almost half of the respondents (44%) experienced at least one practical challenge with 13% experiencing 3 or more. One in five survivors (22%) reported challenges returning to work or school while 21% faced challenges getting to and from appointments and 20% paying for health care. On average, two practical concerns were reported by survivors with at least one concern (see Table 4).

**Table 4:** Prevalence of practical challenges and access to help in adult survey sample

Practical challenges	Number of respondents who answered question	Number of respondents indicating a concern about a practical challenge they experience (mild, moderate, or big)	% of those experiencing a concern about a practical challenge whose concern was ‘*bi*g’	% of those experiencing concern about a practical challenge whose concern was ‘*moderate*’	% of those with a concern about a practical challenge who sought help^a^	% of those who sought help for their concern that experienced difficulty (hard or very hard to find help/no help obtained^a^
Returning to work or school, now or in the future	11,877	2636 (22%)	1076 (41%)	790 (30%)	841 (33%) *N* = 2574	299 (36%) *N* = 836
Getting to and from appointments	12,019	2558 (21%)	476 (19%)	874 (34%)	1031 (43%) *N* = 2388	262 (26%) *N* = 1020
Paying for health care bills	11,958	2402 (20%)	587 (24%)	792 (33%)	818 (37%) *N* = 2227	430 (53%) *N* = 809
Difficulty getting health or life insurance	11,838	1859 (16%)	671 (36%)	529 (28%)	536 (31%) *N* = 1750	355 (67%) *N* = 532
Taking care of children, elders or other family members	11,877	1529 (13%)	364 (24%)	496 (32%)	419 (29%) *N* = 1443	163 (39%) *N* = 414

^a^Note that only those who answered relevant question(s) were included. N refers to the denominator for each concern

Of those who faced practical challenges, returning to work (41%) and getting life insurance (36%) were common issues and about a third sought help. The largest proportion sought help for getting to and from appointments (43%). Of those who sought help, the largest percent who experienced difficulty was getting help obtaining insurance (67%) or paying for health care bills (53%).

Of those who had concerns, 94% indicated the reason they did not ask for help was because they did not want to ask (16%) or they did not know about services available to help them (18%).

### Information availability

The majority of survivors agreed/somewhat agreed that the information they needed was available to them (85%), the information they received was useful (85%), and information about side effects was available (81%). More than half found information about cancer recurrence (62%) and community resources (58%) was available. Overall, more survivors agreed that useful information about physical concerns (75%) was available to them than about emotional (54%) or practical (54%) concerns.

### Unmet need

Table 5 presents the number of respondents concerned about a change and the percent of those individuals who did not receive help, regardless of whether they sought help or not. In total, 7717 individuals identified fatigue as a concern, while 7327 identified anxiety. Overall, more individuals expressed concern about physical changes than emotional and practical ones.

**Table 5:** Summary of unmet needs in adult survey sample

	Number of respondents who were concerned about this need (mild, moderate or big)	Of those who were concerned who did not get help, regardless of whether they sought help
Changes in relationships with friends and colleagues (E)	2371	2081 (88%)
Changes to body image (i.e., confidence in appearance, etc.) (E)	4377	3529 (81%)
Changes in relationships with family, partners (E)	3706	3010 (81%)
Changes to concentration, memory (Ph)	4356	3435 (79%)
Difficulty getting health or life insurance (Pr)	1746	1338 (77%)
Changes in sexual intimacy (E)	4770	3523 (74%)
Taking care of children, elders or other family members (Pr)	1438	1050 (73%)
Anxiety, stress, worry about cancer returning (E)	7327	5216 (71%)
Returning to work or school,, now or in the future (Pr)	2569	1791 (70%)
Paying health care bills (Pr)	2218	1548 (70%)
Fatigue/tiredness (Ph)	7717	5270 (68%)
Depression, sadness, loss of interest in everyday things (E)	4953	3388 (68%)
Changes in sexual activity or function (Ph)	5110	3371 (66%)
Getting to and from appointments (Pr)	2377	1404 (59%)
Nerve problems (Ph)	4149	2327 (56%)
Hormonal, menopause or fertility (Ph)	2834	1552 (55%)
Bladder or urinary problems (Ph)	3971	1819 (46%)
Chronic pain or long term pain (Ph)	3845	1731 (45%)
Lymphedema (Ph)	2624	1091 (42%)
Gastrointestinal problems (Ph)	4110	1689 (41%)

*Ph* physical symptom, *E* emotional issue, *Pr* practical challenge

The proportion of individuals expressing concern but not receiving help was highest in both emotional and practical domains, ranging from a low of 59% (getting to and from appointments) to a high of 88% (relationships with friends and colleagues). Within the physical domain, across all changes, more than 40% of individuals did not receive help for the concerns they experienced.

## Discussion

The Transitions Study is the first time a national survey was conducted. The results provide insight into the experiences of Canadian cancer survivors and a foundation for future program development/policy action. Response rates varied from province to province and provided an opportunity for insight into on-line versus paper-based dissemination, and the use of reminder letters versus telephone calls for future surveys. The final adult sample has a balance of men and women with varied demographic characteristics and provided perspectives on the survivor experience. Overall, respondents rated themselves as experiencing fairly high physical and emotional health as well as experiencing good quality of life. However, there was clear indication that large numbers of individuals expressed physical, emotional, and practical changes that were concerning yet they could not get help. Additionally, percentages of physical and emotional symptoms were higher than reported for the general Canadian population [29].

There was a significant proportion of older adults living with other disease conditions that may include symptoms such as pain, mobility limitations, fatigue, and emotional distress [29]. Isolating symptoms related to cancer may not have been easy for respondents and may have led to over-reporting in some cases. Nonetheless, living with co-morbidities is a reality for these survivors and add to the complexities of recovery and coping with the aftermath of cancer. It implies the need for collaboration between medical specialties.

The types of physical changes respondents reported are mirrored in other needs-based surveys [2, 9, 18–20, 22] and reflect a cross-sectional sample of cancer types, stages, and treatment modalities. Fatigue, cognitive effects, and pain have been reported frequently; however, changes in sexuality and neuropathy are emerging and require attention. Understanding the level of the person’s concern (small, moderate, or big) regarding physical changes offers insight into potential impact on daily life. Survivors become acutely aware of what functions may no longer be possible following the completion of treatment and the reality of physical and emotional changes. The challenge is how to identify these changes early and be prepared to assess and intervene before the changes become insurmountable issues.

Similarly, the emotional changes reported mirror other needs-based studies [3, 13, 18, 20]. The reported levels of anxiety and depression emphasize the magnitude of emotional turmoil that may continue following treatment. The emotional impact of changes in sexual intimacy, body image, and relationships was described less frequently in other studies. It has been argued that these changes emerge more clearly for cancer survivors after treatment is over and efforts are made to return to a ‘normal’ pattern of living. To improve survivor experiences, it may be useful to alert cancer patients to these realities and identify those with concerns as early as possible.

A number of practical issues were identified in previous studies (e.g., travel for appointments, out-of-pockets costs, insurance difficulties, assistance with daily chores) [11, 15, 20]. Return to work or school for cancer survivors has seen an increasing amount of attention in recent years and is a significant challenge for survivors [3, 14, 20]. Given the proportion of older and retired adults in this sample, return to work issues may be under-reported in this study.

The results regarding difficulty in seeking and finding help, and the proportion of individuals who did not receive help (whether they asked or not), revealed that many survivors are not receiving the kind of help that would ease their transition and recovery after cancer treatment. Strategies to identify those at risk for experiencing difficulties, if implemented prior to the time of transition, could offset or mitigate challenges or prevent them from emerging later.

The reasons survivors cited not asking for help were concerning and have implications for practice. Health care providers need to be knowledgeable about the potential for issues to arise and what can be done about them.

The issues that survivors identified and their experience in getting help highlights how survivorship care is organized within cancer programs and in primary care settings. Various models of survivorship care have been developed and evidence about their success is emerging, but uptake and implementation has been slow [24].

This study generated a wealth of data and future steps can include analysis of various subgroups such as age, sex, specific disease site, treatment modality, time since diagnosis, and comorbidity. Additionally, analysis could be completed for each province to inform provincial level policy.

## Study limitations

Several limitations exist. The survey was provided in English and French only and was not offered in a format accessible for those with certain disabilities. The survey was relatively long and likely led to missing data. The results included some data from participants that cannot be validated (i.e., disease status, treatment received). Some respondents did not interpret questions as the research was intended. For example, a proportion of respondents may have misinterpreted “treatment” without considering “surgery” as treatment, or misinterpreted “active surveillance” as follow-up care. Finally, some respondents provided answers that seemed to reflect acute treatment rather than the period following treatment. Future surveys will need to isolate time periods and cancer types.

## Concluding remarks

This study was undertaken to provide a foundation for action. The perspectives of cancer survivors are needed to create services that meet their needs. These general results indicate that many survivors have concerns about physical, emotional, and practical issues yet are not receiving the help to reduce the suffering from their concerns. It is imperative we take action to address this reality.
